# Ten common mistakes that could ruin your enrichment analysis

**DOI:** 10.1371/journal.pcbi.1014122

**Published:** 2026-04-22

**Authors:** Anusuiya Bora, Matthew McKenzie, Mark Ziemann

**Affiliations:** 1 School of Life and Environmental Sciences, Faculty of Science, Engineering and Built Environment, Deakin University, Melbourne, Australia; 2 Burnet Institute, Melbourne, Australia; 3 Institute for Physical Activity and Nutrition, Deakin University, Melbourne, Australia; Montreal, CANADA

## Abstract

Functional enrichment analysis (FEA) is an incredibly powerful way to summarise complex genomics data into information about the regulation of biological pathways including cellular metabolism, signalling and immune responses. About 10,000 scientific articles describe using FEA each year, making it among the most used techniques in bioinformatics. While FEA has become a routine part of workflows via myriad software packages and easy-to-use websites, mistakes can easily creep in due to poor tool design and unawareness among users of pitfalls. Here we outline ten mistakes that undermine the effectiveness of FEA which we commonly see in research articles. We provide practical advice on their mitigation.

## Introduction

PubMed searches indicate keywords like “pathway analysis” and “enrichment analysis” appear in titles or abstracts of approximately 10,000 articles per year, and that number has increased by a factor of 5.4 between 2014 and 2024 (Table A in S1 Supporting Information). The purpose of FEA is to understand whether functional gene categories are differentially represented in the molecular profile at hand, and involves querying hundreds or thousands of gene sets that represent pathways or other functional categories. The versatile nature of FEA means it can be applied on different types of profiling data including proteomics, transcriptomics, genomic variant searches, and chromatin/epigenomics analyses [1,2].

There are a variety of methods for FEA, but two main methods are over-representation analysis (ORA) and functional class scoring (FCS) [3,4]. ORA involves selecting genes based on a hard cut-off followed by a test of enrichment (e.g.,: Fisher’s exact test) as compared to a background list [5]. Popular ORA web tools include DAVID [6], g:Profiler [7] and Enrichr [8], while clusterProfiler [9] is popular for R-based analysis. FCS involves ranking all detected genes followed by a test to assess whether the distribution of scores deviates towards the up- or down-regulated directions. GSEA [10] is a stand-alone FCS software with a graphical user interface, and there are several command-line implementations such as fgsea [11].

Recommendations on correct application of FEA have been previously published [1,4,12–14], yet we and others continue to observe mistakes and methodological deficiencies in peer-reviewed publications at an alarming rate [15,16]. The purpose of this education article is to share what our group has learned about successful FEA over the past 15 years, having authored several articles using it and critically examining hundreds of published articles describing the use of FEA.

Using an example RNA-seq dataset and simulation analysis, we provide evidence to show just how impactful these mistakes are. Details of this analysis are provided in S1 Supporting Information.

### 1. Using uncorrected *p*-values for statistical significance

Enrichment tests generate *p*-values (probability values) between 0 and 1. The *p*-value estimates the probability of an observed enriched category occurring by random chance. A low *p*-value (e.g., *p* < 0.05) suggests the observed result would be unlikely from random data, suggesting a real effect. However, as gene set libraries can contain thousands of categories, we could expect 5% of these to meet the *p* < 0.05 threshold with random data. Therefore, we almost always get many “significant” results just by chance [4]. Our previous literature study showed that this problem was prevalent in 43% of FEA articles [16].

There are a variety of *p*-value correction methods to reduce the risk of false positives [12], including approaches from Sidak [17], Holm-Bonferroni [18] and Benjamini–Hochberg [19]. The Benjamini–Hochberg method, also called the false discovery rate (FDR) method, appears to be the most widely used in genomics to adjust *p*-values, but it has been critiqued as being overly conservative when a larger fraction of tests are not null [20].

Our simulation analysis identified a mean of 10.4 Reactome pathways as significant (*p* < 0.01) from randomly selected foreground genes when *p*-value correction was not implemented; this was reduced to 0.02 pathways, after FDR correction (S1A Fig). Our example analysis indicated that ~45% of pathway enrichment results could be false if correction of *p*-values is not conducted (S1B Fig).

To avoid unacceptable false positives, use a tool that provides adjusted significance values like FDR. *P*-value adjustment can also be done separately with other tools like Stata, SPSS, GraphPad and R. A stricter FDR significance threshold, like 0.01, has been shown to be effective in reducing false positives as compared to 0.05 [21].

### 2. Wrong background gene list

Defining a background list (a.k.a. universe or reference) is crucial because genes that have no chance of being part of the foreground, i.e., undetected genes, should not contribute to enrichment calculations as they will inflate significance values [4,12].

Every type of omics analysis has its limitations. Microarrays can only measure genes they are designed to assay. RNA-seq has certain genes that are poorly detected as a result of sequence similarity or GC bias. Biological differences also play a big part in what is detected [15]. From 78k human genes annotated in Ensembl’s latest release (v115), typically only 12–20k are expressed at detectable levels with RNA-seq in any one tissue.

The severity of this problem is contentious, but our previous analysis of seven RNA-seq studies suggested that using the wrong background could lead to false positive rates of 60%–80% (FDR < 0.05 was used) [16].

The example analysis with a background of detected genes identified 207 significant pathways, but a background of all annotated genes led to an additional 601 false-positive pathways (S2A Fig).

Recommendations for selecting a detection threshold and defining a background list are given in Box 1.

**Box 1. Recommendations for setting a detection threshold to define the background list from various omics data**

Proteomics: Missing values are common. Consider keeping proteins detected in ≥50% of samples.

RNA-seq, scRNA-seq, ChIP-Seq and ATAC-seq: Various valid approaches:

- Mean read count of 10 across all samples.
- Mean reads per million threshold of 1.0 across all samples.
- Read counts of 10 or more in ≥50% of samples.
- Mean reads per million threshold of 1.0 in ≥50% of samples.

Microarray gene expression and DNA methylation: Discard known problematic probes. Include all genes with probes that pass quality control filtering.

Genomics (e.g.,: variant searches by whole genome or exome sequencing): All annotated genes could be used unless there are reasons to believe that some are not detected (e.g., some genes might not be included in the exome enrichment process, extreme GC content, etc.). This can be checked using sequence depth tools.

### 3. Using a tool that does not report enrichment scores

FDR values can tell us whether an observation is statistically significant, but it does not inform whether it is biologically impactful [22,23]. For that, we need some measure of effect size. In FEA, we can use an enrichment score as a surrogate measure of effect size. For rank-based tools like GSEA, the enrichment score varies from -1 to +1, denoting the distribution of genes in a gene set relative to all other genes [10]. For a gene set composed of 15 genes, a score of 1.0 would mean that these 15 genes are the top 15 upregulated, while if a value is close to 0, it means the distribution of genes is close to what you might get by random chance. For over-representation methods like DAVID, the fold enrichment score is often quoted, which is the odds ratio of genes of a gene set in the foreground list as compared to the background [7]. Unfortunately, many common tools do not provide enrichment scores (e.g., clusterProfiler and g:Profiler), which leaves researchers with no information about their effect sizes. Tools that provide enrichment scores include ShinyGO (web) [24], GSEA [10] and fgsea (fora) [11].

### 4. Prioritising results solely by *p*-value

FEA can return hundreds of significant results, which can be confusing to interpret. Many tools by default will sort the results by significance, but this can result in missing the most interesting results. As *p*-value prioritisation emphasises generic functions with large gene sets and moderate fold changes, there is a risk of overlooking smaller gene sets with larger fold changes (contrast Tables B and C in S1 Supporting Information). Smaller and more specific gene sets with larger magnitude enrichment scores are generally better candidates for downstream validation due to their explanatory power.

To avoid this problem, end users should also do enrichment score prioritisation, by first removing pathways above the FDR threshold (e.g.,: 0.05 or 0.01) and then sorting by enrichment score magnitude.

### 5. Foreground lists that are too large or too small for ORA

It is a common misconception that only differentially expressed genes that meet the FDR threshold should be submitted to an enrichment test. Tarca and colleagues suggest a heuristic that selects the top 1% of genes if there are none that meet the standard significance cut-off [25]. If proper FDR control of enrichment results is applied (See #1 above), then gene selection criteria can be flexible. The caveat is that enrichment tests (like the hypergeometric method) have size ranges of input gene lists that work best. If the number of foreground genes is too large, then the enrichment scores will not be as large or interesting, but if the foreground is too small, then the overlap with pathways will be small and fail to reach statistical significance.

Our testing suggests that a gene list size of 700–900 genes, or 5%–9% of all those detected would be optimal for a differential expression study (S4 Fig). To achieve this number, thresholds for significance or fold change filtering can be fine-tuned. Nevertheless, some users may want to avoid setting seemingly arbitrary thresholds—in that case, using an FCS method like GSEA instead that calculates enrichment from all detected genes is recommended.

### 6. Not running ORA separately on up- and down-regulated genes

In some articles, we noticed that authors did not conduct separate ORA tests for up- and down-regulated gene lists, instead opting to submit the combined list for ORA. This is not necessarily an error, as it tests the hypothesis that some pathways are “dysregulated”; a mix of up- and down-regulated genes which appear at an elevated rate. However, the results from the “combined” and “separate” approaches are very different.

The example dataset showed the combined approach identified 82% fewer significant results as compared to the separate approach (S5 Fig). There were no enriched pathways specific to the combined test.

The reason behind this is 2-fold. Firstly, we know that genes in the same pathway are typically correlated with each other [26]. Consider cell cycle genes, or genes responding to pathogens, which are activated in unison to coordinate a complex biological process. In a typical differential expression experiment after a stimulus, this results in pathways that are predominantly up- or down-regulated, but rarely a mix of up and down. Due to this phenomenon, the up and down lists each have relatively strong enrichments, but they are diluted when combined [27]. Based on this, ORA users should use both the combined and separate approaches if directional information is available (some omics types do not).

### 7. Using shallow gene annotations

One of the most important decisions for FEA is selecting the pathway or ontology database to query. There are many options to consider, both proprietary and open source. When choosing, users should consider whether the database contains the gene sets that they a priori suspect will be altered. Secondly, consider the breadth and depth of the pathway library; this will be where the unexpected discoveries may occur and it pays to use a comprehensive library to capture as many aspects of the dataset as possible.

The example analysis showed that using a larger pathway database like Reactome or Gene Ontology Biological Process results in richer results as compared to smaller databases like KEGG (Table D in S1 Supporting Information).

Using a smaller database like KEGG may be justified based on a priori hypotheses, but in most cases where the goal is discovery of novel themes, a larger pathway database would be recommended. Users should be aware that these larger databases have some degree of redundancy which can be confusing to interpret [28].

### 8. Using outdated gene identifiers and gene sets

Data repositories like Gene Expression Omnibus (GEO) [29] contain thousands of previously published data sets that we can reanalyse with new pathway databases and software tools to gain further insights. However, when the data is several years old, we should use it with caution, as many gene names may have changed. For example, Illumina’s EPIC DNA Methylation microarray was released in 2016, and in the following eight years, 3,253 of 22,588 gene names on the chip changed (14.4%) [30]. Therefore, these genes would not be recognised by the FEA software. To update defunct gene symbols, the HGNChelper R package can help [31], also having the benefit of fixing gene symbols corrupted by Excel autocorrect, which are unfortunately common in GEO [32]. Persistent gene identifiers like Ensembl (e.g., ENSG00000180096) and HGNC (e.g., HGNC:2879) are less likely to change over time and are therefore preferable over gene symbols (e.g., SEPTIN1) for FEA.

FEA users should also understand how well updated their preferred pathway databases are. Actively updated databases like Reactome [33] constantly increase in size as annotation consortia continue assimilating functional information from the literature (See S6 Fig). The version of KEGG available on MSigDB has not changed since 2010, but versions available through the KEGGREST API and DAVID Knowledgebase appear to be regularly updated. A regularly updated database is likely to lead to richer and more relevant FEA findings [34].

### 9. Bad presentation

Bad presentation of data is not exclusive to pathway enrichment, but there are a few key mistakes that should be avoided:

1. The number or proportion of selected genes in a category is sometimes shown as evidence of enrichment, but this can be misleading because it does not take into consideration the frequency of these genes in the background list. Enrichment scores and adjusted *p*-values are better for this purpose.
2. Presenting enrichment results as a pie chart is not recommended because it is not possible to show enrichment scores and significance values in this form. Bubble or bar charts are better alternatives.
3. Sometimes a network of genes or pathways are shown, but the significance of nodes and edges are not described.
4. Figures missing key elements such as axis labels.
5. FEA mentioned in the abstract but no data shown in the main article or supplement.
6. Confusion around which tool was used for each figure and panel.

Such misinterpretation and data presentation problems can also occur when a tool is used without understanding the statistical basis of inference [35], so it is crucial that users take the time to familiarise themselves with the tool’s documentation and recommendations.

### 10. Neglecting methods reproducibility

According to Goodman and colleagues [36], methods reproducibility is:

“the provision of enough detail about study procedures and data so the same procedures could, in theory or in actuality, be exactly repeated.”

There are several crucial pieces of information required in order to enable reproducibility of FEA including;

- how genes were selected or scored—especially whether up- or down-regulated genes were considered separately or combined,
- the tool used, and its version,
- the options or parameters applied,
- the statistical test used,
- the gene set/pathway database(s) queries, and their versions,
- for ORA, how a background list was defined, and,
- how *p*-value correction was done [14,37].

A systematic literature analysis published in 2022 found insufficient background list description in 95% of articles describing ORA tests, and *p*-value correction was insufficiently described in 43% of articles, suggesting that FEA generally suffers from a lack of methods reproducibility [16]. Examples of poor and good methods reproducibility are provided in S1 Supporting Information, together with an AI prompt that users could use to assess their Methods sections.

In addition to including the methodological details mentioned above, authors could also provide gene profile data and/or gene lists used for FEA as supplementary files, or better still, provide full reproducibility and transparency with the five pillars framework [38].

## Other issues

There are several more subtle issues not covered in depth here, but are worth mentioning as they have been flagged as potential problems. First, the length of genes is known to impact the ease at which they are detected and so correction of gene length has been suggested to improve enrichment results [39,40]. Second, many FEA tests use genes as the sampling unit and do not take into consideration (or model) biological variation which could yield unrealistic significance values [41]. Third, the size of gene sets, even though they represent similar biology, can disproportionately impact significance scores and complicate interpretation [42,43]. Fourth, tight correlation between each gene’s expression within a pathway could exacerbate false positive rates [26,44]. Fifth, gene sets with a high degree of overlap could be a source of false positives, and enrichment algorithms have been adjusted to circumvent this potential problem [45,46]. It is recommended that users assess the overlap of resulting gene set enrichments using a tool such as “enrichment map” (GSEA) or “clustered heat map” (DAVID) because observed enrichments may be driven by the same subset of genes [4,47]. Sixth, slight differences in the implementation of ORA tests can impact results in some circumstances [48]. Lastly, some web-based FEA tools lack longevity. For example, DAVID version 6.8 [6,49] has been used for over 10,000 publications, but since 2022 has been taken offline, leaving these articles irreproducible. As web-based tools appear to be the most popular option for FEA [16,50], tools that expressly allow preservation/archiving as a Docker image [e.g., 24] are recommended to enable future reproducibility and transparency [51].

## Conclusion

Methodological problems in FEA are likely a combination of poor researcher training, supervision and peer-review scrutiny. The design of tools and (low) quality of tool documentation might also play a role. We also know that inadequate methods have a type of advantage compared to more rigorous ones due to researcher preferences to present “significant” findings [52] and reliance upon default settings even if they are incorrect [16,43]. Problems 2, 3, 5 and 6 appear to be specific to ORA-based tools, and can be avoided entirely by switching to FCS tools like GSEA, which has the added benefit of enhanced accuracy in terms of precision and recall [21,48]. Although learning and running FCS tools is more difficult and time-consuming, the benefits to the quality of results are substantial. A related issue is the overinterpretation (and indeed misinterpretation) of omics data. Researchers should be mindful of the specific biological context of their study, as this directly impacts the interpretation of the results obtained. FEA excels at generating hypotheses but requires separate validation to draw definitive conclusions.

## Supporting information

S1 Supporting Information Additional evidence supporting the recommendations in the main text. This includes information about the analysis conducted, including methods and detailed description of results.
(PDF)

S1 Fig FDR control reduces false positives. **(A)** Simulation analysis demonstrates the effect of FDR control on enrichment results from a set of 1,000 random genes. Box plots show results of 100 simulations. **(B)** Euler diagram demonstrates the impact of FDR correction of *p*-values on the number of ‘significant’ gene sets in the example gene profile (AML3 cells with and without azacitidine exposure). Significance threshold is *p* < 0.01 or FDR < 0.01.
(EPS)

S2 Fig A custom background list is essential for ORA with RNA-seq data. **(A)** Impact of background list selection on the number of significant Reactome gene sets (FDR < 0.01) in the example gene profile (AML3 cells with and without azacitidine exposure). **(B)** Simulation analyses demonstrate the impact of background list selection on the number of ‘significant’ gene sets (FDR < 0.01). The incorrect background includes all genes described in the annotation set, while the correct background includes only the genes that met the detection threshold. 100 simulations. **(C)** Gene sets appearing as false positives in 100/100 simulations include those related to cancer.
(EPS)

S3 Fig Scatterplot showing absolute enrichment scores (x-axis) and log-transformed significance values (y-axis) for each detected pathway. Gene sets with FDR < 0.01 are highlighted in red. If these results are sorted by statistical significance values, then specific pathway enrichments are at risk of being overlooked. Therefore, users should also prioritise results by enrichment score.
(EPS)

S4 Fig Effect of gene list size (x-axis) on number of significant pathways (y-axis). Red and dark blue correspond to significant pathways without filtering on the fold enrichment score (FES). Pink and light blue include pathways that meet the minimum FES of 3.0. Upregulated pathways are shown in red and pink. Downregulated pathways shown in dark blue and light blue. Significance threshold was FDR < 0.01.
(EPS)

S5 Fig Comparison of separate and combined ORA test results. Separate analysis yields many more results at FDR < 0.01 significance level.
(EPS)

S6 Fig Reactome gene set growth over time. Gene sets were downloaded from the MSigDB website, except for 2025_09 which represents the latest gene sets downloaded directly from Reactome but not yet incorporated into MSigDB.
(EPS)
