# Supplementary material for: Ten common mistakes that could ruin your enrichment analysis
Source: PLoS Comput Biol. 2026 Apr 22;22(4):e1014122. doi: 10.1371/journal.pcbi.1014122 (PMC13102193; doi:10.1371/journal.pcbi.1014122)
Supplement: S1 Supporting Information — This includes information about the analysis conducted, including methods and detailed description of results. (PDF) [file pcbi.1014122.s001.pdf]

S1 Supporting Information

Here we provide additional evidence supporting the recommendations in the main text.

Methods

To ascertain the number of functional enrichment analysis (FEA) articles published each year, a PubMed search was conducted using the following search query "(pathway analysis[Title/Abstract]) OR (enrichment analysis[Title/Abstract]) OR (gene ontology analysis[Title/Abstract]".

The example RNA-seq dataset used involves AML3 cells with and without azacytidine treatment [1]. This dataset was selected because it represents a typical transcriptomic study; with three experimental replicates and over 1000 differentially expressed genes. Read counts were obtained from the DEE2 database using the getDEE2 R package using SRA project accession SRP038101 as a query [2]. Genes with average expression above 10 counts per sample were considered detected, and genes not meeting this criterion were removed from downstream analysis (unless stated). Differential expression was conducted using DESeq2 v1.48.2 [3]. Human gene symbols were updated to Ensembl 115 [4]. Genes with FDR<0.01 were considered significantly differentially expressed. Reactome gene sets were downloaded in GMT format on 2nd Sept 2025 [5], and these gene sets were used for all subsequent enrichment tests unless otherwise stated. All ORA tests were conducted using the fora function belonging to the fgsea package v1.34.2 [6]. Minimum gene set size was set to 5 for all subsequent enrichment tests. Gene sets with FDR<0.01 were considered significantly differentially regulated.

To demonstrate the importance of FDR control (Mistake 1), random sampling of 1000 detected genes was followed with ORA with and without FDR control. This was repeated 100 times. For comparison of the nominal and FDR corrected results, the top 1000 up- and 1000 down-regulated genes by p-value altered by azacitidine treatment underwent separate ORA tests using a background consisting of the 13,063 detected genes with gene symbols. The number of significant gene sets (FDR<0.01) from each test were combined for visualisation using an Euler diagram.

To demonstrate the importance of a suitable background gene list (Mistake 2), random sampling of 1000 detected genes as the foreground followed by ORA using either all annotated genes as the background or custom background consisting of detected genes. This process was repeated 100 times. To show the effect of incorrect background on the azacitidine gene profile, 1000 up- and 1000 down-regulated genes selected by p-value underwent ORA using a background including all 55,503 annotated genes. These results were compared to those generated using a background consisting of the 13,063 detected genes using an Euler diagram.

To demonstrate the importance of using enrichment scores for interpretation of FEA results, (Mistake 3 and 4) the mitch package v1.20.0 was used for FCS analysis of the differential expression data [7].

To investigate how foreground gene set size impacts the number of ORA results (Mistake 5), the top N significant up- and down-regulated genes were selected for ORA compared to the background consisting of detected genes, where N was varied between 50 and 7000.

To demonstrate the effect of combining up and down regulated genes into a single test (Mistake 6), the top significant 750 genes irrespective of fold change direction were used as the foreground for an ORA test, and compared with results from the separate approach with 750 up- and 750 down-regulated genes respectively.

To show the effect of using shallow gene annotations (Mistake 7), various gene set libraries were extracted from the MSigDB Collection (msigdb.v2025.1.Hs.symbols.gmt) [8]. These gene set libraries were each used for ORA tests with foreground lists of 750 genes as above.

To show the difference in size of old gene sets (Mistake 8), KEGG and Reactome gene sets were extracted from archived MSigDB Collections going back to 2010. These were compared to the most recent Reactome release (2nd Sept 2025) obtained directly from the Reactome website. KEGG pathway gene sets were also obtained from the KEGG API using the KEGGREST R/Bioconductor package which appears to be updated (Feb 2026).

Instances of poor presentation (Mistake 9) and methods reproducibility (Mistake 10) were drawn from unpublished notes made during a previous systematic analysis of the literature [9].

Analysis was conducted in R 4.5.1 using a Bioconductor Docker image corresponding to Bioconductor release 3.21. Analysis code is available from GitHub (https://github.com/markziemann/10mistakes) and the Docker image is available from DockerHub (https://hub.docker.com/repository/docker/mziemann/10mistakes/general). These will be archived to Zenodo upon acceptance.

Results

FEA keywords in PubMed

The PubMed search revealed 9788 journal articles with the FEA related keywords “pathway analysis”, “enrichment analysis” or “gene ontology analysis” in the title or abstract published in 2024 (Table A). In 2014 it was 1804, so it has increased by a factor of 5.4.

Table A. The number of hits for FEA keywords has drastically increased since 2014. Search conducted 6th Feb 2026.

Year	Count
2025	11500
2024	9788
2023	9135
2022	9903
2021	8170
2020	6278
2019	4660
2018	3652
2017	2965
2016	2459
2015	2209
2014	1805
2013	1496
2012	1178
2011	916
2010	690
2009	513
2008	373
2007	283
2006	163
2005	63
2004	37
2003	22
2002	25
2001	6
2000	7

1. Using uncorrected p-values for statistical significance

Using a simulation approach, we randomly selected 1000 genes that met the detection threshold and submitted these to ORA with gene sets from Reactome. Of the 1840 Reactome gene sets with five or more members detected, on average we obtained a mean of 10.4 hits ($p<0.01$; 100 repeats) with these random genes (S1 Fig panel A), proving that reporting raw p-values is bound to yield some false positives, in this case at a rate of 0.56%. When conducting the same analysis, but using a threshold of FDR<0.01 the mean number of significant pathways was just 0.02.

In the example dataset (AML3 cells with and without azacitidine treatment), there were 2498 differentially expressed genes, (FDR<0.01) with 1066 up- and 1432 down-regulated. The top 1000 up- and down-regulated genes by p-value were selected for separate ORA tests. Omitting FDR correction led to the identification of 380 Reactome pathways with $p<0.01$, of which 173 are likely false positives (45.5%) (S1 Fig panel B).

2. Wrong background gene list

In the example dataset based on an earlier Ensembl release (v90) there are 58,302 annotated genes. Using a minimum detection threshold of ≥10 reads per sample on average across the six samples, only 13,168 genes pass this filter and 45,134 are discarded. Although 77.4% of genes are discarded at this step, they account for a miniscule 0.2% of reads.

We then performed ORA of azacitidine-responsive genes using two different backgrounds. Firstly with a custom background consisting of detected genes, 207 statistically significant sets were identified (FDR<0.01). Secondly, using all annotated genes in the genome as background, yielded 807 significant. There were 206 sets in common between these analyses, giving a Jaccard statistic of 0.20 (S2 Fig panel A).

We then used a simulation approach to examine the consequence of omitting a custom background gene list on an RNA-seq experiment. For the foreground, we drew a random set of 1000 genes in the AML3 example dataset with expression above 10 reads per sample on average and then used an enrichment test (hypergeometric) with all annotated genes as a background, we identified 262 gene sets reaching the FDR<0.01 significance level on average (S2 Fig panel B). For comparison, a custom background gene list composed of genes meeting the 10 reads per sample threshold was used, resulting in 0.02 gene sets with FDR<0.01, practically eliminating false positives. These false positives are highly reproducible (S2 Fig

panel C), and as many of them are cancer-relevant pathways like cell cycle, transcription regulation by TP53 and lipid metabolism, they have the potential to mislead readers.

4. Prioritising results solely by p-value

To demonstrate the problem with p-value prioritisation, see the results from a typical FEA with p-value prioritisation or enrichment score (ES) prioritisation after removal of non-significant pathways (Table B and Table C).

Table B. Top upregulated pathways when prioritised by FDR. This emphasises larger and more generic functions.

**Gene set**	**Set Size**	**p-value**	**FDR**	**ES**	**Mean Log2FC**
Cell Cycle	589	1.4e-47	2.6e-44	0.35	0.069
Metabolism of RNA	725	4.4e-46	4.0e-43	0.31	0.079
Cell Cycle, Mitotic	479	1.5e-39	9.1e-37	0.35	0.066
Cell Cycle Checkpoints	246	2.1e-27	9.6e-25	0.40	0.081
M Phase	336	9.5e-27	2.9e-24	0.34	0.065
Mitotic Prometaphase	189	4.6e-25	1.1e-22	0.44	0.120
Mitotic Metaphase and Anaphase	208	8.9e-24	1.8e-21	0.40	0.110
Mitotic Anaphase	207	2.3e-23	4.3e-21	0.40	0.110
Processing of Capped Intron-Containing Pre-mRNA	273	6.6e-23	1.1e-20	0.35	0.084
Resolution of Sister Chromatid Cohesion	114	1.0e-20	1.6e-18	0.51	0.140

Table C. Top upregulated pathways when prioritised by ES. Pathways with FDR>0.01 were excluded. This emphasises smaller, more specific categories with larger effect sizes.

**Gene set**	**Set Size**	**p-value**	**FDR**	**ES**	**Mean Log2FC**
Activation of NOXA and translocation to mitochondria	5	1.1e-03	6.9e-03	0.84	0.26
Condensation of Prometaphase Chromosomes	11	2.5e-06	3.5e-05	0.82	0.26
Postmitotic nuclear pore complex (NPC) reformation	27	1.8e-11	6.4e-10	0.75	0.21
Phosphorylation of Emi1	6	1.6e-03	8.8e-03	0.75	0.24
Interactions of Rev with host cellular proteins	37	6.8e-15	5.2e-13	0.74	0.20
Nuclear import of Rev protein	34	1.7e-13	9.9e-12	0.73	0.20
Rev-mediated nuclear export of HIV RNA	35	1.0e-13	6.3e-12	0.73	0.20
Transport of Ribonucleoproteins into the Host Nucleus	32	2.1e-12	1.0e-10	0.72	0.20
Export of Viral Ribonucleoproteins from Nucleus	32	2.9e-12	1.3e-10	0.71	0.19
NEP/NS2 Interacts with the Cellular Export Machinery	32	2.9e-12	1.3e-10	0.71	0.19

P-value prioritisation (Table B) emphasises generic functions with large gene sets and moderate fold changes, while enrichment score prioritisation (Table C) highlights smaller gene sets with highly specific functions, where member genes have bigger fold changes.

The scatterplot shown in S3 Fig shows how focusing on statistical significance only could overlook interesting results with larger effect sizes. End users should therefore use both prioritisation approaches to interpret their data.

5. Foreground lists that are too large or too small for ORA

We tested different input gene list sizes in ORA and found that a list of 3000 yielded the most significantly enriched pathways (294 with FDR<0.01), while a small list size of 200 genes yielded only 41 pathways (S4 Fig). In the range of 300-1000 genes, there's a steep increase in the number of significant pathways obtained, with the gradient reducing with more than 1000 genes. This result indicates that using a larger gene list of up to 3000 genes would be better, but the main downside is that these additional statistically significant results correspond to relatively small enrichment scores. For example, significantly downregulated pathways at a gene set size of 100, mean fold enrichment scores were 17.4, and at a size of 3000, they were only 2.2. Users may wish to pre-specify a fold enrichment score that they consider biologically relevant (3-5 seems reasonable), and then tune their analysis to capture the most statistically significant enrichments above this score. In the example dataset, the number of gene sets meeting a minimum fold enrichment score of 3 appeared to peak with a gene set size of 700 for the upregulated genes, and 900 for downregulated genes (S4 Fig), which corresponds to 5-7% of all detected genes.

This suggests that a gene list size of 700-900 genes, or 5-7% of all those detected would be a reasonable recommendation for a differential expression study. Nevertheless, some users may want to avoid setting seemingly arbitrary thresholds - in that case, using an FCS method like GSEA instead that calculates enrichment from all detected genes would be recommended.

6. Not running ORA separately on up and down-regulated genes

In ORA, up- and down-regulated genes can be combined into a single list, or divided into separate lists for separate enrichment tests. We conducted both analyses for the example dataset. The separate approach identified 171 significant pathways (FDR<0.01) compared to

the combined approach that found only 31 (82% fewer; S5 Fig). There were no pathways observed to be uniquely enriched in the combined test.

7. Using shallow gene annotations

Table D compares pathway database size metrics, including the raw number of gene sets, the total number of annotations, the median gene set size and the number of genes with one or more annotations. It shows that KEGG Legacy and KEGG Medicus, available from MSigDB, appear small when compared to the version of KEGG pathways available through the API or DAVID Knowledgebase. Reactome and Gene Ontology's Biological Process (GOBP) are larger still (Table D), although these two pathway resources are known to have some redundancy in gene sets. Consequently, ORA yielded substantially more significant pathways with Reactome and GOBP as compared to KEGG libraries.

Table D. Size metrics of selected gene set libraries, and the number of differentially regulated pathways in the AML3 experiment (FDR<0.01).

	No. gene sets	Total no. annotations	Median gene set size	No. genes with ≥1 annotation	Up-regulated	Down-regulated
KEGG Legacy (MSigDB)	186	12800	52.5	5245	3	23
KEGG Medicus (MSigDB)	658	9662	11.5	2788	2	5
KEGG API (KEGGREST)	369	39991	79.0	9564	6	57
Reactome	1787	97544	23.0	11369	79	45
GOBP	7583	616242	20.0	18000	176	624
MSigDB	35134	4089406	47.0	43351	1600	3766

8. Using outdated gene identifiers and gene sets

The size of Reactome pathway database growth over time is shown in S6 Fig. It shows a constant growth from 430 pathways in 2010 to 2159 pathways in 2025. In contrast, in this period, the size of KEGG Legacy as available on MSigDB has not changed, remaining static at 186 annotated pathways. The version of KEGG as available via the KEGGREST API has 369 pathways, indicating that it is more up to date.

ORA users should be aware that static pathway databases like KEGG Legacy as available on MSigDB will not contain pathways related to newly discovered and annotated molecular pathways as compared to more actively updated databases like KEGG (API) or Reactome. S6

Fig also shows that Reactome underwent two big increases between versions, therefore, users should download and use the newest available version of pathway gene sets.

9. Bad presentation

To support the examples of bad presentation mentioned in the main text, here are the citations.

1. The number of selected genes in a category is often shown as evidence of enrichment, [15–18], but this can be misleading because this is one of four numbers that goes into calculating a fold enrichment score. Similarly, the proportion of selected genes that belong to a gene category is sometimes shown [19–22], but this does not directly reflect the fold enrichment score.
2. Presenting enrichment results as a pie chart [16,21,23,24] is not recommended because it is not possible to show enrichment scores and significance values in this form.
3. Sometimes a network of genes or pathways are shown, but the significance of nodes and edges are not described [25].
4. Figures missing key elements such as axis labels [16,26–29].
5. FEA mentioned in the abstract but no data shown in the main article or supplement [30,31].
6. Confusion around which tool was used for each figure and panel [32].

Lastly it is worth mentioning that excessive use of tools and databases can make the results difficult to interpret, as in [24].

10. Neglecting methods reproducibility

A previous literature analysis [9] identified some inadequate methodological descriptions which are worth including here as they illustrate the dire state of methods reproducibility:

“GO and KEGG enrichment analysis were performed on the DEGs by perl scripts in house.” [33].

“Gene ontology (GO) and pathway analysis were performed in the standard enrichment computation method.” [34].

We've also noted some cases where FEA was not described in the methods at all, despite being important enough to mention said results in the abstract [35−37]. Moreover, we've identified cases where the tool mentioned in the methods section is inconsistent with what's shown in the results [38,39].

Box 2 shows excerpts from recent transcriptome articles using ORA and FCS that we think are good examples.

Box 2. Positive examples of FEA methods as applied to RNA-seq for ORA and FCS adapted from Ziemann et al, (2024) and Bastawy et al, (2024). Some changes were made to conform with the recommendations in this article.

ORA: *For each transcriptome dataset, kallisto (v0.43.1) (Bray et al. 2016) transcript counts were aggregated to the gene level. Genes with fewer than 10 reads per sample on average were removed from downstream analysis. The remaining genes passing this selection were included in the background gene set. Differential expression analysis was conducted with DESeq2 v1.44.0 (Love et al. 2014), with genes identified by their Ensembl identifiers. Gene symbols were then fetched using biomaRt v2.60.0, based on Ensembl version 112 (Durinck et al. 2009). Human gene sets were obtained from MSigDB v2023.2 (Liberzon et al. 2015). Foreground genes were defined as the top 700 top statistically significant differentially expressed genes , with separate lists for up- and down-regulated genes. These gene lists were subjected to ORA using clusterProfiler's enricher function v4.12.0, using a minimum set size of 5 and no maximum set size. This function uses a hypergeometric test to ascertain p-value estimates of enrichment. Enrichment results with FDR<0.01 were considered significant.*

FCS: *The reference mouse transcriptome was downloaded from GENCODE version 28 [144]. The raw reads (FASTQ) were inspected for sequence quality using Fastqc (v0.11.9) [145] and trimmed using Skewer (v0.2.2) [146] to remove Q < 20 bases from 3' ends. Kallisto (0.46.1) was used to align the paired-end RNA-seq reads to the mouse transcriptome [147]. The counts at the transcript level were imported into RStudio v4.1 and then consolidated into counts at the gene level. Genes displaying an average count of less than 10 reads across samples were omitted from subsequent analysis. The differential gene expression between control and asthmatic groups was performed using the generalized linear model proposed and implemented in the R package DESeq2 (1.36.0). DESeq2 is one of the most popular statistical tools for differential expression analysis. It makes full use of biological replicate information to estimate differential expression p-values and effect sizes, as detailed in the publication by Love et al. [148]. Volcano plots and heatmaps were generated in base R. For pathway analysis, REACTOME gene sets were obtained from the Molecular Signatures Database and converted to mouse gene identifiers with the msigdbr R package (1.4.0) [149,150,151]. Differential pathway analysis was then performed with the mitch R package (1.8.0) with default settings [152]. Mitch is a functional class scoring technique that uses a rank–ANOVA test to ascertain the collective enrichment of genes in either the up- or downregulated direction. To reduce the chance of false positives, differential gene and pathway analyses were subjected to false discovery rate (FDR) correction using the method of [153]. Genes and pathways with an FDR < 0.01 were considered statistically significant. All the data were analyzed using RStudio version (2022.07.1) and GraphPad prism version 8 software.*

Box 3 contains a prompt that could be provided to an AI chatbot to identify some of the most crucial methodological deficiencies for ORA and FCS analyses.

Box 3. Example AI chatbot prompts for examining key methodological details in ORA and FCS based FEA.
ORA: I need you to carefully examine how pathway enrichment analysis was conducted in this scientific article. I need you to focus on the Methods section, but information can also be found in other sections. Please answer the following questions separately, so that the output data can be collated into a table. If authors did not provide any details in the article, then please write 'not described'.<br>1. What tool was used for pathway enrichment analysis?<br>2. Was a tool version number provided?<br>3. What gene set library was queried (eg: GO, KEGG, Reactome or other)?<br>4. How was the foreground gene list selected for pathway enrichment analysis?<br>5. Was a background gene list defined for pathway enrichment analysis?<br>6. What statistical test was used for enrichment analysis?<br>7. Was false discovery rate correction used to control the number of false positives in the pathway enrichment analysis?<br><br>FCS: I need you to carefully examine how pathway enrichment analysis was conducted in this scientific article. I need you to focus on the Methods section, but information can also be found in other sections. Please answer the following questions separately, so that the output data can be collated into a table. If authors did not provide any details in the article, then please write 'not described'.<br>1. What tool was used for pathway enrichment analysis?<br>2. Was a tool version number provided?<br>3. What gene set library was queried (eg: GO, KEGG, Reactome or other)?<br>4. Was a detection threshold applied to the data?<br>5. How were genes ranked prior to enrichment analysis?<br>6. What statistical test was used for enrichment analysis?<br>7. Was false discovery rate correction used to control the number of false positives in the pathway enrichment analysis?

Bibliography

1. Lund K, Cole JJ, VanderKraats ND, McBryan T, Pchelintsev NA, Clark W, et al. DNMT inhibitors reverse a specific signature of aberrant promoter DNA methylation and associated gene silencing in AML. Genome Biol. 2014;15: 406.

2. Ziemann M, Kaspi A, El-Osta A. Digital expression explorer 2: A repository of uniformly processed RNA sequencing data. Gigascience. 2019;8: giz022.

3. Love M, Anders S, Huber W, et al. Differential analysis of count data–the DESeq2 package. Genome Biol. 2014;15: 10–1186.

4. Dyer SC, Austine-Orimoloye O, Azov AG, Barba M, Barnes I, Barrera-Enriquez VP, et al. Ensembl 2025. Nucleic Acids Res. 2025;53: D948–D957.

5. Milacic M, Beavers D, Conley P, Gong C, Gillespie M, Griss J, et al. The reactome pathway knowledgebase 2024. Nucleic Acids Res. 2024;52: D672–D678.

6. Korotkevich G, Sukhov V, Budin N, Shpak B, Artyomov NM, Sergushichev A. Fast gene set enrichment analysis. bioRxiv. 2021. doi:10.1101/060012

7. Kaspi A, Ziemann M. Mitch: Multi-contrast pathway enrichment for multi-omics and single-cell profiling data. BMC Genomics. 2020;21: 447.

8. Liberzon A, Birger C, Thorvaldsdóttir H, Ghandi M, Mesirov JP, Tamayo P. The molecular signatures database hallmark gene set collection. Cell Systems. 2015;1: 417–425.

9. Wijesooriya K, Jadaan SA, Perera KL, Kaur T, Ziemann M. Urgent need for consistent standards in functional enrichment analysis. PLoS Comput Biol. 2022;18: e1009935.

10. Sullivan GM, Feinn R. Using effect size—or why the p value is not enough. Journal of Graduate Medical Education. 2012;4: 279–282.

11. Schober P, Bossers SM, Schwarte LA. Statistical significance versus clinical importance of observed effect sizes: What do p values and confidence intervals really represent? Anesthesia & Analgesia. 2018;126: 1068–1072.

12. Subramanian A, Tamayo P, Mootha VK, Mukherjee S, Ebert BL, Gillette MA, et al. Gene set enrichment analysis: A knowledge-based approach for interpreting genome-wide expression profiles. Proc Natl Acad Sci USA. 2005;102: 15545–15550.

13. Tilford CA, Siemers NO. Gene set enrichment analysis. Methods Mol Biol. 2009;563: 99–121.

14. Ge SX, Jung D, Yao R. ShinyGO: A graphical gene-set enrichment tool for animals and plants. Bioinformatics. 2020;36: 2628–2629.

15. Zhao Z, Zhao Q, Zhu S, Huang B, Lv L, Chen T, et al. iTRAQ-based comparative proteomic analysis of cells infected with eimeria tenella sporozoites. Parasite. 2019;26: 7.

16. Li C, E C, Zhou Y, Yu W. Candidate genes and potential mechanisms for chemoradiotherapy sensitivity in locally advanced rectal cancer. Oncology Letters. 2019;17: 4494–4504.

17. Sarıman M, Abacı N, Ekmekçi SS, Çakiris A, Paçal FP, Üstek D, et al. Investigation of gene expressions of myeloma cells in the bone marrow of multiple myeloma patients by transcriptome analysis. Balkan Medical Journal. 2019;36: 23.

18. Bhatia G, Sharma S, Upadhyay SK, Singh K. Long non-coding RNAs coordinate developmental transitions and other key biological processes in grapevine. Scientific Reports. 2019;9: 3552.

19. Wang X, Diao L, Sun D, Wang D, Zhu J, He Y, et al. OsteoporosAtlas: A human osteoporosis-related gene database. PeerJ. 2019;7: e6778.

20. Wang X-M, Tian F-Y, Fan L-J, Xie C-B, Niu Z-Z, Chen W-Q. Comparison of DNA methylation profiles associated with spontaneous preterm birth in placenta and cord blood. BMC Medical Genomics. 2019;12: 1.

21. Hu F, Li Y, Yu K, Huang B, Ma X, Liu C, et al. ITRAQ-based quantitative proteomics reveals the proteome profiles of primary duck embryo fibroblast cells infected with duck tembusu virus. BioMed Research International. 2019;2019: 1582709.

22. Koh SY, Moon JY, Unno T, Cho SK. Baicalein suppresses stem cell-like characteristics in radio-and chemoresistant MDA-MB-231 human breast cancer cells through up-regulation of IFIT2. Nutrients. 2019;11: 624.

23. Liu Y, Zhu D, Xing H, Hou Y, Sun Y. A 6-gene risk score system constructed for predicting the clinical prognosis of pancreatic adenocarcinoma patients. Oncology Reports. 2019;41: 1521–1530.

24. Lin Y-T, Wu P-H, Tsai Y-C, Hsu Y-L, Wang HY, Kuo M-C, et al. Indoxyl sulfate induces apoptosis through oxidative stress and mitogen-activated protein kinase signaling pathway inhibition in human astrocytes. Journal of Clinical Medicine. 2019;8: 191.

25. Bandi S, Tchaikovskaya T, Gupta S. Hepatic differentiation of human pluripotent stem cells by developmental stage-related metabolomics products. Differentiation. 2019;105: 54–70.

26. Boyko AV, Girich AS, Eliseikina MG, Maslennikov SI, Dolmatov IY. Reference assembly and gene expression analysis of apostichopus japonicus larval development. Scientific Reports. 2019;9: 1131.

27. Lou W, Ding B, Fan W. High expression of pseudogene PTTG3P indicates a poor prognosis in human breast cancer. Molecular Therapy-Oncolytics. 2019;14: 15–26.

28. Shi Y, Sun H, Wang X, Jin W, Chen Q, Yuan Z, et al. Physiological and transcriptomic analyses reveal the molecular networks of responses induced by exogenous trehalose in plant. PLoS One. 2019;14: e0217204.

29. Li M, Guo Y, Feng Y-M, Zhang N. Identification of triple-negative breast cancer genes and a novel high-risk breast cancer prediction model development based on PPI data and support vector machines. Frontiers in Genetics. 2019;10: 180.

30. Xu L, Wang L, Zhou L, Dorfman RG, Pan Y, Tang D, et al. The SIRT2/cMYC pathway inhibits peroxidation-related apoptosis in cholangiocarcinoma through metabolic reprogramming. Neoplasia. 2019;21: 429–441.

31. Di Gerlando R, Mastrangelo S, Sardina MT, Ragatzu M, Spaterna A, Portolano B, et al. A genome-wide detection of copy number variations using SNP genotyping arrays in braque français type pyrénées dogs. Animals. 2019;9: 77.

32. Jin L, Zhu C, Qin X. Expression profile of tRNA-derived fragments in pancreatic cancer. Oncology Letters. 2019;18: 3104–3114.

33. Zhou T, Luo X, Yu C, Zhang C, Zhang L, Song Y, et al. Transcriptome analyses provide insights into the expression pattern and sequence similarity of several taxol biosynthesis-related genes in three taxus species. BMC Plant Biology. 2019;19: 33.

34. Liu F, Wei J, Hao Y, Tang F, Jiao W, Qu S, et al. Long noncoding RNAs and messenger RNAs expression profiles potentially regulated by ZBTB7A in nasopharyngeal carcinoma. BioMed Research International. 2019;2019: 7246491.

35. Hu N, Cheng Z, Pang Y, Zhao H, Chen L, Wang C, et al. High expression of MiR-98 is a good prognostic factor in acute myeloid leukemia patients treated with chemotherapy alone. Journal of Cancer. 2019;10: 178.

36. Zhao J, Xu J, Chen B, Cui W, Zhou Z, Song X, et al. Characterization of proteins involved in chloroplast targeting disturbed by rice stripe virus by novel protoplast–chloroplast proteomics. International Journal of Molecular Sciences. 2019;20: 253.

37. Chen L, Chen Q, Kuang S, Zhao C, Yang L, Zhang Y, et al. USF1-induced upregulation of LINC01048 promotes cell proliferation and apoptosis in cutaneous squamous cell carcinoma by binding to TAF15 to transcriptionally activate YAP1. Cell Death & Disease. 2019;10: 296.

38. Li M, Li A, Zhou S, Lv H, Yang W. SPAG5 upregulation contributes to enhanced c-MYC transcriptional activity via interaction with c-MYC binding protein in triple-negative breast cancer. Journal of Hematology & Oncology. 2019;12: 14.

39. Tong Y, Song Y, Deng S. Combined analysis and validation for DNA methylation and gene expression profiles associated with prostate cancer. Cancer Cell International. 2019;19: 50.
